# Toll-Like Receptor 6 differential expression in two pig genetic groups vaccinated against *Mycoplasma hyopneumoniae*

**DOI:** 10.1186/1753-6561-5-S4-S9

**Published:** 2011-06-03

**Authors:** Katiene Régia Silva Sousa, André Mauric Frossard Ribeiro, Paulo Roberto Nunes Goes, Simone Eliza Facioni Guimarães, Paulo Sávio Lopes, Renata Veroneze, Eliane Gasparino

**Affiliations:** 1 Departamento de Zootecnia – Universidade Federal de Viçosa, Viçosa, Minas Gerais, Brasil, 36570-000; 2 Departamento de Zootecnia – Universidade Estadual de Maringá, Maringá, Paraná, Brasil, 87020-900

## Abstract

**Background:**

*Mycoplasma hyopneumoniae* is the etiologic agent of enzootic pneumonia, which causes important economic losses to swine industry. The Toll-like receptors (TLRs) are pattern-recognition receptors which detect microbial presence and initiate the innate as well as the adaptative immune defense. Toll-like receptor 6 is a type I transmembrane protein that recognizes bacterial components. The aim of this study was to compare mRNA expression pattern of TLR6 gene in two genetically distinct groups of pigs vaccinated against *Mycoplasma hyopneumoniae.*

**Methods:**

For each genetic group, peripheral blood was collected just before and 10 days after vaccination from 10 Naturalized Brazilian Piau breed and 10 Commercial White Line serum-negative female piglets. RNA was extracted from peripheral blood mononuclear cells (PBMCs), reverse transcripted and the qRT-PCR performed using SYBR green fluorescence system, using GAPDH gene as endogenous control. Analyses were performed by UNIVARIATE (Shapiro-Wilk test) and MIXED procedures of SAS software (version 9.0).

**Results:**

It was observed significant interaction between breed and vaccination, being the TLR6 mRNA expression higher in the Commercial White line than in the Piau breed after vaccination. Furthermore, there was differential expression before and after vaccination in the Commercial White line.

**Conclusions:**

Analysis of in TLR6 gene expression showed difference between the two distinct genetic groups, however, other TLRs gene expression must be evaluated for a better understanding of innate resistance in the pig concerning *Mycoplasma hyopneumoniae* infection.

## Background

*Mycoplasma hyopneumoniae* (Mhp) is the primary agent of porcine enzootic pneumonia, a chronic respiratory disease endemic to pig farms characterized by high morbidity and low mortality rates; it occurs worldwide and causes major economic losses to the pig industry as it causes increased susceptibility to secondary respiratory infections due to the *M. hyopneumoniae*-associated deactivation of mucociliary functions [7]. In vertebrates, Toll-like receptors (TLRs) have been identified and their function in innate immunity intensively analyzed [1]. The extracellular regions of TLRs are involved in recognition of pathogen-associated molecular patterns (PAMPs), and the specificity of this recognition is determined by the respective TLR molecules. Hence, their function is important in swine infectious diseases [8]. Toll-like receptor 6 (TLR6) is a type I transmembrane protein that recognizes *Mycoplasma hyopneumoniae* pathogen derived molecules patterns [4].

Differences in production of immune molecules such as TLRs have a deep influence on response to pathogens and they are associated with disease resistance and susceptibility [5]. Studies related to transcriptomic analysis front of antigen in specific population as well as involving local and domestic breeds have been accomplished to discover differential expressed genes involved in the innate and adaptive immune response. The local breeds, for example, Naturalized Brazilian Piau breed has peculiar characteristics as rusticity, adaptability to poor conditions of management and feeding, and a great resistance to diseases [3], which make them proper for studies aiming to improve the knowledge concerning host-pathogen interaction and susceptibility/resistance diseases.

Thus, the aim of this study was to examine the expression of TLR6 in two genetically distinct groups of pigs, Brazilian naturalized Piau breed and a Commercial White line, vaccinated against *Mycoplasma hyopneumoniae* in order to better understand some of the molecular mechanisms involved in protective immunity and inflammatory reactions against this pathogen.

## Methods

### Porcine peripheral blood mononuclear cells (PBMCs) collection

Twenty female piglets seronegative for Mhp (10 Brazilian Naturalized Piau breed and 10 Commercial White line) from the pig farm at the Federal University of Viçosa (Viçosa, MG, Brasil) were utilized. Blood samples from *sinus orbitalis* were collected just before and 10 days after vaccination into tubes containing EDTA. PBMCs were isolated by density gradient centrifugation using Ficoll-Paque Plus (GE Healthcare) of 1,077 g/mL density.

### RNA isolation and cDNA syntesis

The total RNA was extracted from PBMCs using RNeasy® Mini Kit protocol (Qiagen), and DNAse digested according to manufacter’s instructions. RNA yield and quality were assessed using the NanoVue Plus spectrophotometer (GE Healthcare) and Agilent 2100 BioAnalyzer (Agilent Technologies), respectively. Equivalents amounts of RNA from PBMCs were reverse transcribed with SuperScript III/RnaseOut Enzyme Mix (Invitrogen Life Thecnologies) to evaluated gene expression before and after vaccination against Mhp.

### Real Time PCR

Quantitative real-time PCR was performed using SYBR green fluorescent detection system ABI Prism 7300 Sequence Detection Systems (Applied Biosystems). Reactions were performed using 100 ng of the cDNA and 100 nM and 400 nM primer for TLR6 and GAPDH genes, respectively. The thermal cycling conditions were 40 cycles of 30 s melting at 95^?^ C followed by 30 s of annealing and extension at 60^?^ C. The linearity of amplification for TLR6 was similar to the endogenous control gene (GAPDH), data not shown. Gene expression data was presented using ΔCt values.

### Statistical analysis

The results were analyzed using UNIVARIATE (Shapiro-Wilk test) and MIXED procedures of SAS software (version 9.0) in order to evaluate the data distribution and perform the variance analysis and the contrast comparisons, respectively.

## Results and discussion

Gene expression in response to bacterial infection can be the result of many factors that modulate the amount and timing of bacteria reaching each tissue or organ, and these factors may differ among individuals and genetic lines. The data had normal distribution (Shapiro-Wilk test, P<0.05). Gene expression results are shown in Table 1.

**Table 1:** TLR6 expression levels between breeds were normalized to the expression of GAPDH (mean ΔCt ± standard error).

	Piau	Commercial
Before vaccination	5,60±0,37^Aa^	5,01±0,34^Aa^
After vaccination	5,29±0,35^Aa^	3,59±0,34^Bb^

Different letters indicate significant differences of mean values between breeds (A and B) (P<0.001) and within breeds (a and b) (P<0.02).

In terms of ΔCt, TLR6 expression was significantly influenced by breed (P<0.003). The level of TLR6 gene expression significantly differed after vaccination between breeds (P<0.001), being highest in the Commercial White line, since it showed a decrease in the ΔCt value, which means an increase of gene expression in response to the agent; suggesting that Commercial line has higher sensibility to vaccination. In addition, there was significant effect before and after vaccination in the Commercial line (P<0.02).

TLR6 has been associated with altered signalling and cytokine production as well as differences in susceptibility/resistance to diseases [7]. Domestic animals enable analysis of populations exposed to environmental challenges and different selection criteria. Moreover, comparisons to local breeds can shed light on the evolutionary process [2] regarding TLR6 activity. The present study shows that the local, not genetically improved breeds have potential to elucidate genetic differences in resistance/susceptibility response to Mhp. In this regard, the comparison between Commercial White line and the Piau breed showed that it can be informative in elucidate the role of host defense against Mhp in swine. More work in being done by our group to better understand the role of the TLRs in the immune response of the pig.

## Conclusion

This result shows difference in TLR6 gene expression between the genetic groups, however, we need to know much more about the integrated responses that occur when intact bacteria infects the host. This can be done improving the understanding of the genetic and molecular mechanisms that regulate the pig immune response to pathogens.

## Competing interests

The authors declare that they have no competing interests.

## Authors' contributions

KRSS, AMRF, PRNG and PSL carried out the molecular genetics studies. SEFG supervised the researches. EG and RV performed the statistical analyses. KRSS, RV and SEFG drafted the manuscript. All authors read and approved the final manuscript.
